# Trends in receiving chemotherapy for advanced cancer patients at the end of life

**DOI:** 10.1186/s12904-015-0001-7

**Published:** 2015-03-13

**Authors:** Hee Seung Lee, Kyeong Hyeon Chun, Dochang Moon, Hahn Kyu yeon, Sanghoon Lee, SooHyeon Lee

**Affiliations:** Division of Medical Oncology, Department of Internal Medicine, Yonsei University College of Medicine, Seoul, Republic of Korea

**Keywords:** End of life, Advanced cancer, Chemotherapy, Palliative care

## Abstract

**Background:**

The use of chemotherapy in advanced cancer patients has increased with the development of novel, high-efficacy anticancer therapeutic agents. In the current study, we analyzed the 10-year trends in patients receiving chemotherapy at the end of life.

**Method:**

We retrospectively reviewed mortality data for advanced cancer patients who died in 2000, 2005, and 2010 at a single institution. The trends of receiving palliative chemotherapy at the end of life were assessed for each year. In addition, logistic regression analysis was performed to determine the factors associated with receiving chemotherapy.

**Results:**

We analyzed the records of 2,345 patients who died of cancer. Patients with less responsive tumors were less likely to receive chemotherapy than patients with responsive tumors at the time of death. Patients who were ≥ 65 years were less likely to receive chemotherapy compared with patients who were < 65 years at the end of life. However, the proportion of older patients receiving chemotherapy in the last month of life increased in 2010 (44.2%) compared with 2005 (32.7%) and 2000 (25.7%). Compared with the year 2000, the likelihood of receiving chemotherapy during the last 1 month of life increased in 2005 (odds ratio [OR], 2.05; 95% confidence interval [CI], 1.60–2.62) and 2010 (OR, 4.42; 95% CI, 3.51–5.57).

**Conclusions:**

The proportion of patients receiving chemotherapy at the end of life increased successively from 2000 to 2005 to 2010. Physicians should consider whether to continue chemotherapy at the end of life.

## Background

Novel, high efficacy anticancer therapeutic agents have been developed in the last decade [1-6]. The availability of new anticancer agents has prolonged the timeline of chemotherapy use in cancer patients [1]. In particular, the use of chemotherapy at the end of life (EOL) in advanced cancer patients has increased [7], and EOL care has become increasingly aggressive [8]. In a study by Liu et al. [7], the rates of continued chemotherapy during the last month of life increased from 17.5% in 2001 to 21.0% in 2006. Previous studies also demonstrated that the proportion of indicators for aggressive EOL care, including receiving EOL chemotherapy, increased significantly in the last 10 years [6]. Many advanced cancer patients receive EOL chemotherapy, even those with cancer types known to be unresponsive to chemotherapy [9]. However, in some studies, patients who received palliative chemotherapy during the last month of life had a significantly shorter survival time from the beginning of palliative treatment to death and more frequent hospital admissions [6,10,11].

Recognition of the importance of high quality EOL care has continued to increase. However, few studies have included recent data and the use of oral chemotherapeutic agents. Therefore, we evaluated the trends in EOL chemotherapy in advanced cancer patients who died between 2000 and 2010 in a single institution in the Republic of Korea to confirm recent trends in receiving EOL chemotherapy.

## Methods

### Study population

We analyzed the trends in receiving EOL chemotherapy during a recent 10-year period at Severance Hospital, a tertiary referral hospital in the Republic of Korea that does not have an inpatient hospice unit. We retrospectively reviewed the records of advanced cancer patients who died at Severance Hospital in 2000, 2005, or 2010 to determine the number of patients who received chemotherapy for 6, 3, and 1 month before death. The exclusion criteria were as follows: 1) < 18 or > 90 years old (*n* = 65), 2) uncertainty regarding the time and regimen of the last chemotherapy (*n* = 20), and 3) died in a location other than Severance Hospital (*n* = 80). The study protocol conformed to the ethical guidelines of the 1975 Declaration of Helsinki. The protocol of this study was approved by the institutional review board (4-2014-1054) at Severance Hospital and written informed consent for this study was not required by the institutional review board because the researchers only accessed the database for analysis purposes and personal information was not used.

Patient information was collected from electronic medical records, including age, gender, date of diagnosis, marital status, place of residence, job, date of death, and type of cancer. The patients were divided into groups according to age (younger vs. older than 65 years) and place of residence based on a previous study [12]. Patients were also grouped by type of cancer, because some studies suggested that selectivity in the EOL chemotherapy regimen was based on the responsiveness of the cancer type. Specifically, breast, stomach, lung, ovarian, and colon cancers tend to be more responsive to chemotherapy, whereas melanoma, gallbladder, pancreatic, and hepatocellular cancer tend to be less responsive to chemotherapy [9,13-16]. Finally, the type of chemotherapy was characterized according to intravenous or oral administration.

### Statistical analyses

Chi-squared tests were performed to evaluate potential differences in patients who received versus did not receive chemotherapy in each of the years evaluated. Univariate logistic regression analyses were performed to evaluate whether each factor affected the number of individuals who received EOL chemotherapy in each month prior to death. Then, multivariate logistic regression analyses were performed after adjusting for factors that were significant in the univariate regression analysis to identify the factors related to the number of patients receiving EOL chemotherapy in each month prior to death. Data were analyzed using SPSS version 20.0 (SPSS, Chicago, IL, USA), and *P* < 0.05 was considered to be statistically significant.

## Results

### Patient characteristics

A total of 2,510 patients were eligible for inclusion in the current study. The final analysis included 2,345 patients who died at Severance Hospital in 2000, 2005, or 2010, with 601, 702, and 1,042 deaths in each respective year. The 601 patients who died in 2000 included 393 (65%) males and 208 (35%) females, with a median age of 58 (range, 18–89) years. The records of the 702 patients who died in 2005 included 440 (62%) males and 262 (38%) females, with a median age of 61 years (range 18–88). Finally, the 1042 patients who died in 2010 included 685 (65%) males and 357 (35%) females, with a median age of 62 years (range 19–90). No differences were observed in the gender proportions between 2000 and 2010 (65% males vs. 35% females). The use of chemotherapy at the EOL was significantly associated with younger age, the chemosensitivity of the cancer type, and the use of oral chemotherapeutic agents. The use of chemotherapy at the EOL was higher in males, but not significantly. Table 1 shows the baseline characteristics of the advanced cancer patients who received EOL chemotherapy.

**Table 1:** **Characteristics of the cancer patients who received or who did not receive chemotherapy during the last months of life**

**Year of death**	**2000**						**2005**						**2010**					
**Months before death**	**6 months**		**3 months**		**1 month**		**6 months**		**3 months**		**1 month**		**6 months**		**3 months**		**1 month**	
Number	CTx (+)	*P* value	CTx (+)	*P* value	CTx (+)	*P* value	CTx (+)	*P* value	CTx (+)	*P* value	CTx (+)	*P* value	CTx (+)	*P* value	CTx (+)	*P* value	CTx (+)	*P* value
	201 (33)		152 (25)		136 (22)		325 (46)		283 (40)		260 (37)		597 (57)		497 (47)		582 (56)	
Gender																		
Male	132 (65.7)	NS	98 (64.5)	NS	83 (61.0)	NS	199 (61.2)	NS	199 (61.2)	NS	165 (63.5)	NS	371 (62.1)	0.005	314 (3.2)	NS	383(65.8)	NS
Female	69 (34.3)		54 (35.5)		53 (39.0)		126 (38.8)		126 (38.8)		95 (36.5)		226 (37.9)		183 (36.8)		199 (34.2)	
Age																		
≥65 years	31 (15.4)	<0.001	29 (19.1)	0.002	35 (25.7)	0.005	83 (25.5)	<0.001	83 (25.5)	<0.001	85 (32.7)	<0.001	232 (38.9)	<0.001	197 (39.6)	0.004	257 (44.2)	NS
<65 years	170 (84.6)		123 (80.9)		101 (74.3)		242 (74.5)		242 (74.5)		175 (67.3)		365 (61.1)		300 (60.4)		325 (55.8)	
Place of residence																		
City	144 (71.6)	NS	111 (73.0)	NS	87 (64.0)	NS	241 (74.2)	NS	241 (74.2)	NS	194 (74.6)	NS	412 (69.0)	NS	351 (70.6)	NS	410 (70.4)	NS
Other	57 (28.4)		41 (27.0)		49 (36.0)		84 (25.8)		84 (25.8)		66 (25.4)		185 (31)		146 (29.4)		172 (29.6)	
Chemosensitivity																		
Others	51 (25.4)	NS	44 (28.9)	NS	53 (39.0)	0.045	86 (26.5)	0.006	86 (26.5)	0.006	99 (38.1)	<0.001	178 (29.8)	0.01	149 (30.0)	0.003	192 (33.0)	0.002
Sensitive	82 (40.8)		63 (41.4)		42 (30.9)		166 (51.1)		166 (51.1)		116 (44.6)		299 (50.1)		255 (51.3)		268 (46.0)	
Insensitive	68 (33.8)		45 (29.6)		41 (30.1)		73 (22.5)		73 (22.5)		45 (17.3)		120 (20.1)		93 (18.7)		122 (21.0)	
Chemotherapy																		
Oral CTx	10 (5.0)	<0.001	14 (9.2)	<0.001	17 (12.5)	<0.001	20 (6.2)	<0.001	20 (6.2)	<0.001	26 (10)	<0.001	138 (23.1)	<0.001	125 (25.2)	<0.001	89 (15.3)	<0.001
IV CTx	191 (95.0)		138 (90.8)		119 (87.5)		305 (93.8)		305 (93.8)		234 (90)		459 (76.9)		372 (74.8)		493 (84.7)	

Variables are expressed as n (%).

NS, not significant.

### Proportion of patients receiving EOL chemotherapy

**Figure 1:**
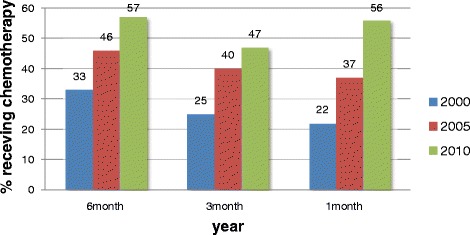
**The proportion of patients receiving chemotherapy at the end of life in 2000,** **2005,** **and 2010.**

**Figure 2:**
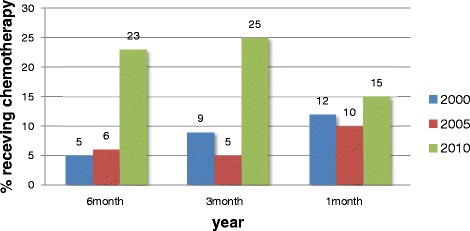
**The proportion of patients receiving oral chemotherapeutic agents in 2000,** **2005,** **and 2010.**

More than half of the patients in 2010 received EOL chemotherapy, and the use of EOL chemotherapy increased in each year evaluated (Figure 1). The proportion of patients receiving chemotherapy during the last month of life increased from 22% in 2000 to 56% in 2010. The percentage of patients receiving chemotherapy during the last 6 months of life increased from 33% in 2000 to 57% in 2010 (*P* < 0.001). In addition, the number of patients who received oral chemotherapeutic agents increased between 2000 and 2010 (Figure 2).

Univariate analyses revealed that age was significantly associated with receiving EOL chemotherapy. Patients < 65 years were more likely to receive chemotherapy during the last 6, 3, and 1 month of life compared with older patients (*P* < 0.001). The proportion of patients aged ≥ 65 years receiving chemotherapy in the last month of life was 44.2% in 2010, compared with 32.7% in 2005 and 25.7% in 2000. Patients with cancers that were relatively chemo-sensitive were more likely to receive chemotherapy during the last month of life in 2000 and 2005 compared with those with chemo-insensitive cancers (*P* = 0.045 and *P* < 0.001, respectively). The proportion of patients who received oral chemotherapeutic agents at the EOL increased in 2010 (*P* < 0.001).

Table 2 shows the OR of receiving chemotherapy according to the year of death using multivariate analysis. Compared with patients receiving chemotherapy in 2000, those in 2005 and 2010 were more likely to receive chemotherapy during the last 6, 3 or 1 month of life (*P* < 0.001). The OR for receiving chemotherapy in the last month of life was 2.05 (95% CI, 1.60–2.62) in 2005 and 4.42 (95% CI, 3.51–5.57) in 2010 compared with 2000. The difference in OR between 2010 and 2000 was greater for receiving chemotherapy during the last month of life compared with the last 6 months of life.

**Table 2:** **Odds ratios of receiving chemotherapy in 2005 and 2010 compared with 2000**

	**6 months**	**3 months**	**2 months**	**1 month**
	OR (95% CI)	OR (95% CI)	OR (95% CI)	OR (95% CI)
2000	Ref	Ref	Ref	Ref
2005	1.94 (1.53–2.43)	2.17 (1.70–2.77)	1.97 (1.54–2.51)	2.05 (1.60–2.62)
2010	3.26 (2.61–4.06)	3.08 (2.45–3.87)	3.50 (2.79–4.39)	4.42 (3.51–5.57)
*P* for trend	<0.001	<0.001	<0.001	<0.001

*OR*, odds ratio; *CI*, confidence interval.

Adjusted for age, gender, chemosensitivity, and route of administration.

## Discussion

Physicians are increasingly considering the importance of patient quality of life at the EOL. In patients with advanced cancer, anticancer treatments aim to improve the quality of life and overall survival. The current study revealed serial changes in EOL chemotherapy. Nappa et al. found no differences in age, gender, or chemotherapeutic agent among patients who did or did not receive chemotherapy within 1 month of death. However, other studies found differences in age, cancer type, cancer chemo-sensitivity, and gender in patients who received chemotherapy at the EOL [12,16-18]. The current study revealed a significant association between EOL chemotherapy and age, gender, cancer chemo-sensitivity, and use of oral chemotherapeutic agents. Males were more likely to receive chemotherapy at the EOL than were females. Consistent with this, a previous study reported that males had a stronger preference for life-sustaining treatment compared with females [19]. Cancer chemo-sensitivity was also a significant factor for the administration of chemotherapy at the EOL, although it was less important recently after the introduction of targeted therapeutic agents. In addition, older patients received EOL chemotherapy less frequently than did younger patients. However, patients older than 65 years have received chemotherapy increasingly as survival times increases.

The overall rates of chemotherapy given within 1 month of death were similar between the current and previous studies. Previous studies reported rates of chemotherapy within 1 month of death in advanced cancer patients of 18–43% [1,10,12,20]. Yun et al. found that chemotherapy was given to 48.7% of patients in the last 6 months, 43.9% in the last 3 months, and 30.9% in the last month of life [12]. The current study found that a high proportion of cancer patients received chemotherapy in the last month of life, and that this proportion increased over a recent 10-year period. Several reasons might explain the increasing use of EOL chemotherapy. First, various novel chemotherapeutic agents have been developed recently, including oral chemotherapeutic agents. This has increased the opportunity for advanced cancer patients to receive palliative chemotherapy. Second, the insurance system was changed in Korea in 2009, which decreased the financial burden for advanced cancer patients. Before these changes, the national health insurance system covered 80% of the total medical fee; however, since 2009 it has covered 95% of the total medical fees. This systemic change might have also affected the trends toward receiving EOL chemotherapy. In addition, attitudes toward EOL treatment are different culturally between Korea and Western countries [21,22]. In Korea, there is a cultural trend of continuing palliative chemotherapy at the EOL rather than receiving hospice care. The hospice system is also not well-developed or established in Korea.

Aggressive cancer care is not necessarily wrong for patients at the EOL. For example, such care might fit with the preferences of advanced cancer patients who want to continue potentially life-prolonging treatment. However, previous studies showed that aggressive palliative chemotherapy at the EOL decreases the quality of life and can increase mortality. Similarly, Keam et al. found shorter survival times and more frequent hospital admissions among patients who received chemotherapy at the EOL [11]. Overly aggressive EOL chemotherapy is recognized as poor quality cancer care [23,24]. Previous studies also showed that ceasing aggressive cancer treatment earlier and introducing palliative care might increase the survival time and quality of life of advanced cancer patients [25]. Therefore, physicians should consider the risks and benefits of continuing palliative chemotherapy at the EOL to allow patients to die with dignity.

The present study has several unique strengths compared with previous studies. First, to our knowledge, this is the largest series to assess EOL chemotherapy in advanced cancer patients. Although one previous study analyzed a large number of patients between 2001 and 2006 [7], the current study evaluated a longer period (from 2000 to 2010) and divided EOL into three lengths of time. The identification of serial changes according to years and the last months before death has increased our understanding of the trends in receiving chemotherapy at the EOL. Second, in contrast to previous reports the current study included patients who received oral chemotherapeutic agents, because these novel targeted agents are now available and are convenient for elderly patients. Finally, we analyzed the effect of the health insurance system on the prevalence of receiving chemotherapy in the Republic of Korea.

Nevertheless, this study has some limitations. The data might have been overestimated because we did not include patients who died at locations other than the hospital who potentially did not select for chemotherapy or were discharged for supportive care. In addition, direct comparisons with earlier studies are difficult, because the current study was performed at a single tertiary hospital. In addition, care at teaching hospitals is likely to be more aggressive [24]. Nevertheless, these data confirm recent treatment trends in advanced cancer patients, because most advanced cancer patients tend to visit large-volume hospitals after cancer diagnosis. Finally, we considered only the proportion of patients who received chemotherapy as an indicator of aggressive treatment. Various factors related to aggressive treatment affect EOL care in patients with advanced cancer. Despite these limitations, the results of the current study are informative. There have been few previous reports related to palliative care using chemotherapy and the use of oral chemotherapeutic agents in patients at the EOL.

## Conclusion

The proportion of patients receiving chemotherapy at the EOL increased from 2000 to 2005 to 2010. Although the data could have been overestimated, physicians should consider whether to continue chemotherapy at the EOL. In a future study, we will review other information including ER visits, hospital admission, ICU admission, and medical costs to evaluate treatment aggressiveness.
